# Book Reviews

**Published:** 1897-06

**Authors:** 


### Book Reviews.

---

Twentieth Century Practice. An International Encyclopedia of Modern Medical Science. By leading authorities of Europe and America. Edited by Thomas L. Stedman, M.D., New York City. In twenty volumes. Volume IX. Diseases of the Digestive Organs. New York: William Wood & Company. 1897.

Whoever may take even a cursory glance at this volume of the important series called the Twentieth Century Practice of Medicine will be impressed with its value, while a more complete examination will lead to the conviction that it is one of the best, if not the very best, in the group thus far published. The authors are distributed over a wide range of territory, compassing almost three-quarters of the globe, reaching from Chicago to Naples and Berlin, thence to Johannesburg in the South African Republic.

Beginning with local diseases of the mouth, we find it to be an interesting chapter, written jointly by Mikulicz and Kümmel, of Breslau. These authors lay special stress on malignant diseases of the soft parts and are especially rich in their pathology. Diseases of the intestines, by Ewald, of Berlin, takes the reader through an absorbing field that is discoursed upon by a most competent author. We have lately expressed our opinion of a treatise on the stomach from the same source. The next section, written jointly by Gibney and Walker, of New York, is devoted to the consideration of hernia and contains some new points that are the subject of illustration. One is ventral hernia following incision after appendicitis; another is congenital lumbar hernia.

Reaching the central portion of the book, we find a section devoted to diseases of the spleen, prepared by Alfred Stengel, of Philadelphia, the distinguished young pathologist, who lately visited Buffalo and read a paper before the Alumni Association of Buffalo University Medical College. Stengel has intelligently grouped all the knowledge we possess in relation to this obscure organ. In the next section Semmola and Gioffredi, of Naples, consider diseases of the liver and, as might be suspected, they appropriate about one-third of the volume to the subject. It is a fruitful field, since almost everybody has a liver and only a few think it healthy. Doctors, too, encourage the belief by charging every obscure or unrecognised symptom to this old-fashioned organ that is generally more sinned against than sinning. Nevertheless, these two well-known writers have succeeded in telling us some new facts in regard to it and have presented them in very readable fashion.

A section in which diseases of the gall-bladder are dealt with is most satisfactorily and intelligently handled by John B. Murphy, of Chicago. This distinguished surgeon has had a large experience in this field, and since it is essentially a surgical subject his selection to write upon it was most fortunate. Finally, the subject of movable kidney is presented by Franks, of Johannesburg, South African Republic, which is an interesting essay.

Volume X. was published some time ago and was reviewed in May, 1897, page 788, so that now one-half the series is completed.

---

Seventeenth Annual Report of the State Board of Health of New York. With fourteen maps of sewer systems and sewage disposal works (in separate volume). Transmitted to the legislature, February 17, 1896. Albany: Wynkoop Hallenbeck Crawford Company, State Printers. 1896.

These reports are among the most interesting official documents issued from the capital at Albany. They deal with questions that have an important bearing upon the health and prosperity of the people and should be studied by sanitarians and public health officers. The board is now presided over by one of the most capable officers it has ever had, and the work done by the board, if he can have his way, will always be a credit to the Empire state. The tuberculosis committee has continued its work and its report indicates some progress in investigating this important field. The efficacy of tuberculin as a diagnostic agent is reaffirmed as the result of further experience by the committee. It says: "By the use of this agent some animals, advanced in the disease, fail to show any reaction, and in other cases animals not as extensively affected will show the highest reaction. In the examinations thus far made not a single error has occurred, as proven by the autopsies."

The protection of water supplies of the smaller cities in the interior is another important field in which this board is doing excellent work.

---

Transactions of the Eighteenth Annual Meeting of the American Laryngological Association, held in the City of Pittsburg, Pa., May 14, 15 and 16, 1896. Henry L. Swain, M. D., Secretary. New York: D. Appleton & Co. 1897.

This annual volume represents the work of one of the oldest and best of the American special societies. The president, Dr. W. H. Daly, in his annual address refrained from wearisome discourse on difficult questions of science, but instead gave some interesting personal reminiscences and talked to the Fellows as though they were members of one family. Among other things he alluded to the fact that when he began to practise laryngology, some twenty years ago, there was scarcely a laryngoscope in use west of the Alleghany mountains. Though he is a student and practiser of laryngology he has yet never ceased to be somewhat of a general practitioner, for he believes that one cannot be a good specialist in diseases of the nose, ear and throat without large experience and knowledge of general medicine. The good sense of this statement will be apparent to every trained physician. When a man starts fresh from college, or even after one or two years general practice, he is a specialist for the general public to avoid.

The volume is replete with interesting papers and able discussions. It is a book that must naturally be found on the table of every laryngologist and those who practise general medicine will do well to obtain it.

---

Lectures on Appendicitis and Notes on Other Subjects. By Robert T. Morris, A. M., M. D., Fellow of the New York Academy of Medicine, American Association of Obstetricians and Gynecologists, American Medical Association; Member of the New York State and County Medical Societies, Society of Alumni of Bellevue Hospital, Linnean Society of Natural History, etc. Second edition, revised and enlarged. With illustrations by Henry Macdonald, M. D. New York: G. P. Putnam's Sons. London: The Knickerbocker Press. 1897.

In our review of the first edition of this monograph (Volume XXXV. of the Journal, page 443, new series,) we expressed a high appreciation of the work of this distinguished author on the lines herein laid down. That the profession in general has shown a like appreciation of the book is indicated by the fact that a second edition is demanded in a little more than a year. It is refreshing to read Morris's rules for surgical cleanliness, or rather his ideas governing that important preliminary to all surgical procedures. What he says on the subject is so simple, so precise and yet withal so imperative, that it is difficult to understand how even the novitiate could fail to comprehend it or to recognise its absolute necessity.

With regard to the subject proper, appendicitis, there is nothing further to add on this occasion. Morris deals with it in a masterful manner. His monograph is one that merits study and it will be quoted as a part of the literature of the subject in years to come; in other words, the principles and practice laid down are such as will stand the test of time. Measured by his results, Morris must be given place in the front rank as an abdominal surgeon.

THE DISEASES OF THE STOMACH. By Dr. C. A. EWALD, Extraordinary Professor of Medicine at the University of Berlin ; Director of the Augusta Hospital, etc. Translated and edited, with numerous additions, from the third German edition, by Morris Manges, A. M., M. D., Assistant Visiting Physician to Mount Sinai Hospital ; Lecturer on General medicine at the New York Polyclinic, etc. Second revised edition. New York: D. Appleton & Co. 1897.

The first American edition of this work was noticed in the JOURNAL, Volume XXXII. New Series, page 314, December, 1892, in which we called attention to a number of its important features. Since that time considerable progress has been made in the knowledge of diseases of the stomach and several other important works have been issued. This has rendered necessary a new American edition, of this one which is based upon the third German edition. This is by the same translator as the former one and he has followed the author's text with few exceptions, but has incorporated much new material which appears either in foot notes or inclosed in brackets. Ewald is recognised as an accurate observer and he is quoted in the literature of diseases of the stomach in almost every tongue. He is especially strong in his pathology, particularly in malignant diseases and ulcer of the stomach. While in this direction there has been of late much increase of pathological knowledge there has not been, it must be confessed with some degree of regret, a corresponding progress in our therapeutic resources. However, whatever has been done that is of value will be found correctly set forth in Ewald's treatise. It has become a recognised text-book, as was predicted when it first appeared, and must be consulted by those clinicians and teachers who desire to keep pace with the most substantial literature.

